# *CHEK2* represses breast stromal fibroblasts and their paracrine tumor-promoting effects through suppressing SDF-1 and IL-6

**DOI:** 10.1186/s12885-016-2614-5

**Published:** 2016-08-02

**Authors:** Maha A. Al-Rakan, Siti-Faujiah Hendrayani, Abdelilah Aboussekhra

**Affiliations:** 1 Present address: Department of Clinical Laboratory Sciences, College of Applied Medical Sciences, King Saud University, Riyadh, 11211 Kingdom of Saudi Arabia; 2 Department of Molecular Oncology, King Faisal Specialist Hospital and Research Center, MBC# 03, PO BOX 3354, Riyadh, 11211 Kingdom of Saudi Arabia

**Keywords:** Breast cancer, Cancer-associated fibroblasts, CHEK2, IL-6, SDF-1

## Abstract

**Background:**

Active fibroblasts, the predominant and the most active cells of breast cancer stroma, are responsible for tumor growth and spread. However, the molecular mediators and pathways responsible for stromal fibroblast activation, and their paracrine pro-carcinogenic effects are still not well defined. The *CHEK2* tumor suppressor gene codes for a protein kinase, which plays important roles in the cellular response to various genotoxic stresses.

**Methods:**

Immunoblotting, quantitative RT-PCR and Immunofluorescence were used to assess the expression of *CHEK2* in different primary breast fibroblasts and in tissues. The effect of *CHEK2* on the expression and secretion of SDF-1 and IL-6 was evaluated by immunoblotting and ELISA. The WST-1 colorimetric assay was used to assess cell proliferation, while the BD BioCoat Matrigel invasion chambers were utilized to determine the effects of *CHEK2* on the migratory and the invasiveness capacities of breast stromal fibroblasts as well as breast cancer cells.

**Results:**

We have shown that *CHEK2* is down-regulated in most cancer-associated fibroblasts (CAFs) as compared to their corresponding tumor counterpart fibroblasts (TCFs) at both the mRNA and protein levels. Interestingly, *CHEK2* down-regulation using specific siRNA increased the expression/secretion of both cancer-promoting cytokines SDF-1 and IL-6, and transdifferentiated stromal fibroblasts to myofibroblasts. These cells were able to enhance the proliferation of non-cancerous epithelial cells, and also boosted the migration/invasion abilities of breast cancer cells in a paracrine manner. The later effect was SDF-1/IL-6-dependent. Importantly, ectopic expression of *CHEK2* in active CAFs converted these cells to a normal state, with lower migration/invasion capacities and reduced paracrine pro-carcinogenic effects.

**Conclusion:**

These results indicate that *CHEK2* possesses non-cell-autonomous tumor suppressor functions, and present the Chk2 protein as an important mediator in the functional interplay between breast carcinomas and their stromal fibroblasts.

## Background

Breast tumors, like other types of solid tumors, are composed of cancer cells as well as various types of stromal cells that constitute the tumor microenvironment [1]. Fibroblasts are the most abundant and active stromal cells, which exhort cancer cells all over the various carcinogenic steps. Cancer-associated fibroblasts (CAFs)-related pro-carcinogenic effects are mediated through paracrine factors, which are under the control of several tumor suppressor genes such as p16 and p53 [2–4]. In addition to their cell-autonomous tumor suppressor function, these proteins possess also non-cell-autonomous tumor suppressive effects that they manifest from stromal fibroblasts [2].

*CHEK2* is another tumor suppressor gene, which is implicated in the pathogenesis of various types of sporadic tumors and is a low penetrance-predisposing gene to sarcoma, brain tumors and familial breast cancer [5]. The two most studied breast cancer predisposing variants of the *CHEK2* gene are the 100delC deletion in the kinase domain in exon 10, and the 470 T > C (I157T) missense mutation in the fork-head-associated (FHA) domain in exon 3. These 2 mutations are associated with approximately 2- fold increased risk of breast cancer [5–7]. A novel recurrent *CHEK2* Y390C mutation has been recently identified in high-risk Chinese breast cancer patients. This mutation impairs *CHEK2* activity and is associated with increased breast cancer risk [8].

*CHEK2* is a multiorgan cancer susceptibility gene that encodes a multifunctional serine/threonine protein kinase. *CHEK2* enables the link between ATM/ATR kinases and downstream checkpoint effectors such as p53 during DNA-damage response [9]. When activated Chk2 phosphorylates various proteins involved in cell cycle regulation, DNA repair, p53 signaling and apoptosis [9]. Furthermore, *CHEK2* plays also a major role in the senescence-associated secretory phenotype (SASP). Indeed, the expression of several SASP-related cytokines, particularly the inflammatory cytokines IL-6 and IL-8, is under the control of a pathway involving *CHEK2* [10]. Therefore, in addition to its capital role in maintaining genomic integrity and preventing fixation of potentially carcinogenic mutations, *CHEK2* is also involved in regulating cellular communication with its microenvironment. Like senescent cells, cancer-associated fibroblasts have also a secretary phenotype responsible for their procarcinogenic effects [11, 12]. Therefore, we sought to investigate the potential role of *CHEK2* in the secretory phenotype of breast stromal fibroblasts and their activation.

We have shown that *CHEK2* inhibits the procarcinogenic effects of breast stromal fibroblasts and has a non-cell-autonomous tumor suppressive function through repressing the expression/secretion of SDF-1 and IL-6.

## Methods

### Cells, cell culture and chemicals

Breast fibroblast cells were obtained, characterized and cultured as previously described [13]. Breast tissues were obtained from patients who underwent surgery at the King Faisal Specialist Hospital and Research Center. Signed informed consent was obtained from all the patients under the Research Ethical Committee Project number RAC#2031091. While CAFs derived from tumors, TCFs were developed from histologically normal tissues located at least 2 cm away from tumors (invasive ductal carcinomas). Processing of breast cancer tissues was performed after routine examination by certified anatomical pathologist using hematoxilin and eosin (HE)-stained sections. NBF-1 cells were developed from healthy age-matched female who performed breast reduction surgery. In the present experiments CAFs and their corresponding TCFs were always cultured simultaneously, in the same conditions and at similar passages (4–8). MDA-MB-231and MCF-10A cell lines were obtained from ATCC and were authenticated before purchase by their standard short tandem repeat DNA typing methodology, and were routinely tested for the presence of the relevant markers, and were cultured following the instructions of the company. All supplements were obtained from Sigma (Saint Louis, MO, USA) except for antibiotics and antimycotics solutions, which were obtained from Gibco (Grand Island, NY, USA). Cells were maintained at 37 °C in humidified incubator with 5 % CO_2_.

Anti-SDF-1 (MAB310) and IgG (6-101-C-ABS) from R&D systems; anti-IL-6 (17901) from Sigma, USA. Blocking antibodies were used at 2.5 μg/mL.

### RNA purification and quantitative RT-PCR

Total RNA was purified using the TRI reagent (Sigma) and single stranded complementary DNA (cDNA) was obtained from reverse transcription of 1 μg of RNA using RT-PCR kit (BD Biosciences) and following the manufacturer protocol. cDNA was then amplified with 1U Taq polymerase, dNTPs (50 mM), and primers (25 pmol each). For real time RT-PCR Syber green and platinum Taq polymerase (Invitrogen) were used and the amplifications were performed utilizing the Bio-Rad iQ5 multicolor Real time PCR detection system. All reactions were performed in triplicates and the data were analyzed using the 2(−Delta Delta C(T)) method [14, 15].

The primers are:

- *CHEK2*: 5′-TGTCCCTCCCAAACCAGTAGTTGT-3′ and 5′-TTCACAGCCCCATGGCAGCG-3′
- *IL-6*: 5′-GACAAAGCCAGAGTCCTTCAGAGA-3′ and 5′-CTAGGTTTGCCGAGTAGATCT-3′
- *GAPDH*: 5′-GAGTCCACTGGCGTCTTC-3′ and 5′-GGGGTGCTAAGCAGTTGGT-3′
- *SDF1*: 5′-TAGTCAAGTGCGTCCACGA-3′ and 5′-GGACACACCACAGCACAAAC-3′

### Cell proliferation assay

Cells were seeded into 96-well plates at 0.5-1.10^4^/well and incubated overnight. The medium was replaced with SFCM and incubated for different time intervals (0, 24 and 48 h). Cell proliferation was measured by the tetrazolium salt WST-1 colorimetric assay, as recommended by the manufacturer (Roche Diagnostics GmbH, Mannheim, Germany). The WST-1 reagent was added to each well, and the plates were then incubated for 4 h at 37 °C. The amount of formazan was quantified using ELISA reader at 450 nm of absorbance.

### Cellular lysate preparation and immunoblotting

This has been performed as previously described [16]. Antibodies directed against alpha smooth muscle actin (α-SMA), Stromal-derived factor-1 (SDF-1), Twist-1, Vimentin (RV202), interleukin-6 (IL-6) and N-cadherin were purchased from Abcam (Cambridge, MA); E-cadherin (24E10), EpCam (UV1D9), Chk2 (2662) from Cell Signaling (Danvers, MA); p53 (DO-1) and Glyceraldehydes-3-phosphate dehydrogenase (GAPDH, FL-335) was purchased from Santa Cruz (Santa Cruz, CA).

### ELISA assays

Supernatants from 24 h fibroblast cell cultures were harvested, and ELISA was performed according to the manufacturer’s instructions (R&D Systems). The OD was used at 450-nm on a standard ELISA plate-reader. These experiments were performed in triplicates and repeated at least twice.

### Chemotaxis and invasion assay

The 24-well BD BioCoat Matrigel Invasion Chambers were used as per the manufacturer guideline (BD Bioscience). 2–4 × 10^5^ cells were added to the upper wells separated by an 8 μm pore size PET membrane with a thin layer of matrigel basement membrane matrix (for invasion) or without (for migration). The membranes were stained with Diff Quick stain (Fisher Scientific) after removing the non-migrated cells from the top of the membrane with Q-tips. After air-drying, the membranes were cut and mounted on slides with oil, and cells that had migrated to the underside of the filter were counted using light microscope (Zeiss Axio Observer) in five randomly selected fields (magnification; 40x). Each assay was performed in triplicate.

### siRNA transfection

*CHEK2*-siRNA (SIH600921A) was obtained from SABiosciences, and the transfection was performed using lipofectamin (Invitrogen) as recommended by the manufacturer.

### Viral infection

Lentivirus based vectors bearing *CHEK2*-ORF and the control (Origene, RC212127L1) were used to prepare lentiviral supernatant from 293FT cells. Lentiviral supernatants were collected 48 h post-transfection, filtered and used for infection. 24 h later, media were replaced with complete medium and cells were grown for 3 days.

### Conditioned media

Cells were cultured in serum-free medium for 24 h, and then medium was collected and centrifuged. The resulting supernatants were used either immediately or were frozen at −80 °C until needed.

### Quantification of protein expression level

Protein signal intensity of each band was determined using ImageQuant TL Software (GE Healthcare). Next, dividing the obtained value of each band by the values of the corresponding internal control allowed a correction of the loading differences.

### Statistical analysis

Statistical analysis was performed by student’s t-test (two tailed and unpaired) and *p*-values < 0.05 were considered as statistically significant.

## Results

### *CHEK2* is down-regulated in cancer-associated fibroblasts

**Fig. 1:**
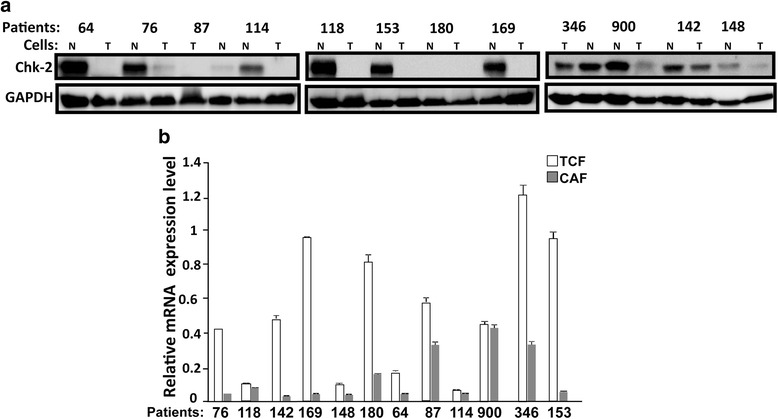
*CHEK2* expression is down-regulated in cancer-associated fibroblasts. **a** Whole cell lysates were prepared from the indicated cells and 50 μg of proteins were used for immunoblotting analysis using antibodies against the indicated proteins. **b** Total RNA was extracted from the indicated cells and the amount of the *CHEK2* mRNA was assessed by qRT-PCR. Error bars represent means ± S.D

We started the present study by assessing the level of the Chk2 protein in CAFs and their corresponding TCFs (tumor counterpart fibroblasts) isolated from the same patients. To this end, we prepared whole cell lysates from 12 CAF/TCF pairs, and then we performed immunoblotting analysis using specific Chk2 antibody and GAPDH was used as internal control. Figure 1a shows that while Chk2 was undetectable in both CAF-180 and TCF-180 cells, the level of the protein was reduced in all the other CAFs as compared to their corresponding TCF cells. The level of Chk2 was also very low in TCF-87 and TCF-148 (Fig. 1a). Next, we assessed the expression of the *CHEK2* mRNA level in CAFs and their counterpart TCFs. Total RNA from the same 12 CAF/TCF pairs was purified and the *CHEK2* mRNA was amplified by quantitative RT-PCR (qRT-PCR) using specific primers, and *GAPDH* was used as internal control. Figure 1b shows that the *CHEK2* mRNA level was lower in 11 out of 12 CAFs (92 %) as compared to their corresponding TCF cells. On the other hand, *CHEK2* mRNA level was similar in TCF-900 and CAF-900 cells (Fig. 1b). Therefore, Fig. 1a and b shows that, in general, there is correlation between the mRNA and protein expression levels among the CAF/TCF pairs. For 3 pairs TCF/CAF-118, TCF/CAF-180 and TCF/CAF-900 there was no correlation between the mRNA and protein levels of *CHEK2*. This shows that *CHEK2* is down-regulated in most CAFs as compared to their corresponding TCFs at both the mRNA and protein levels.

### *CHEK2* induces p53 but represses the expression of α-SMA, SDF-1 and IL-6 in breast stromal fibroblasts

**Fig. 2:**
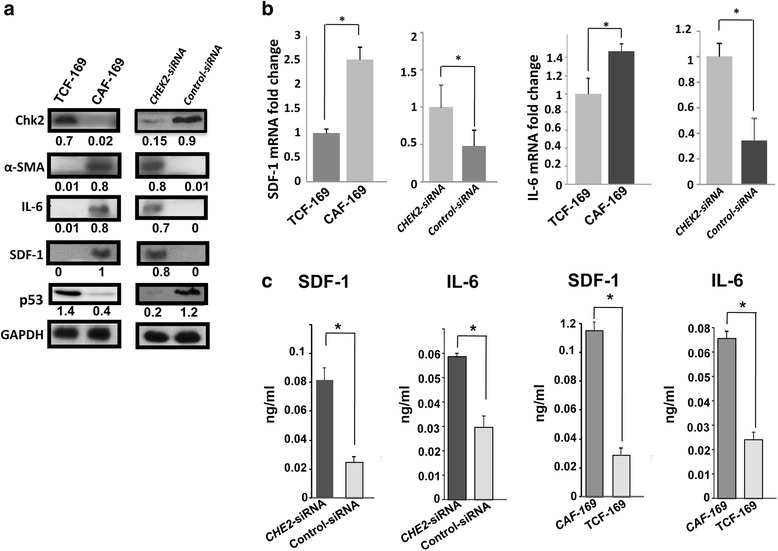
*CHEK2* represses the expression/secretion of SDF-1 and IL-6*.* **a** Whole cell lysates were prepared from the indicated cells and were used for immunoblotting analysis. The numbers below the bands indicate the corresponding expression levels after loading correction against GAPDH. **b** Total RNA was extracted from the indicated cells and used to assess the mRNA levels of SDF-1 and IL-1 using qRT-PCR and specific primers. **c** Conditioned media from the indicated cells were collected after 24 h and the levels of the indicated proteins were determined by ELISA, and were presented in the respective histograms. Error bars represent means ± S.D, *, *p* value < 0.0001

After showing the reduced level of *CHEK2* in CAFs as compared to their respective TCFs from the same patients, we sought to investigate the potential effect of *CHEK2* specific down-regulation on the activation of these cells. To this end, we knocked-down *CHEK2* in TCF-169 cells and the obtained results were compared with those of the corresponding CAF-169 cells. Figure 2a shows efficient down-regulation (6 fold) of Chk2 in TCF-169 by *CHEK2*-siRNA as compared to the corresponding control. This mirrors the expression of Chk2 in TCF-169 and their corresponding CAF-169 cells (Fig. 2a). Interestingly, while the 3 markers α-SMA, IL-6 and SDF-1 were undetectable in TCF-169 and control-siRNA cells, they were strongly up-regulated upon *CHEK2* down-regulation, reaching levels similar to those observed in CAF-169 (Fig. 2a). On the other hand, *CHEK2* knock-down down-regulated p53 (6 fold) as compared to control cells, reaching a level similar to that observed in CAF-169 cells (Fig. 2a). Next, we assessed the effect of *CHEK2* down-regulation on the expression of SDF-1 and IL-6 at the mRNA levels using qRT-PCR. Figure 2b shows 2 fold increase in the level of SDF-1 and 3 fold up-regulation of IL-6 in TCF-169-*CHEK2*-siRNA cells as compared to controls. This parallels the levels of SDF-1 and IL-6 in TCF-169 and their corresponding CAF-169 cells (Fig. 2b). This suggests that *CHEK2* represses the expression of these genes in breast stromal fibroblasts, indicating that *CHEK2* down-regulation may represent an important step towards the activation of these cells.

### *CHEK2* represses the secretion of SDF-1 and IL-6 from breast stromal fibroblasts

To further show the effect of *CHEK2* down-regulation on the expression of important secreted cytokines, we assessed the effect of *CHEK2* Knock-down on the secretion of SDF-1 and IL-6. To this end, serum-free conditioned media (SFCM) was collected from TCF-169-*CHEK2*-siRNA and their corresponding control cells, and then the secreted levels of SDF-1 and IL-6 was assessed by ELISA. Figure 2c shows that the down-regulation of *CHEK2* increased the secretion of SDF-1 and IL-6 by 3.3 and 2 fold, respectively. This mirrors the secreted levels of SDF-1 and IL-6 in TCF-169 and their corresponding CAF-169 cells (Fig. 2c).

### *CHEK2* represses the migration/invasion abilities of breast stromal fibroblasts

**Fig. 3:**
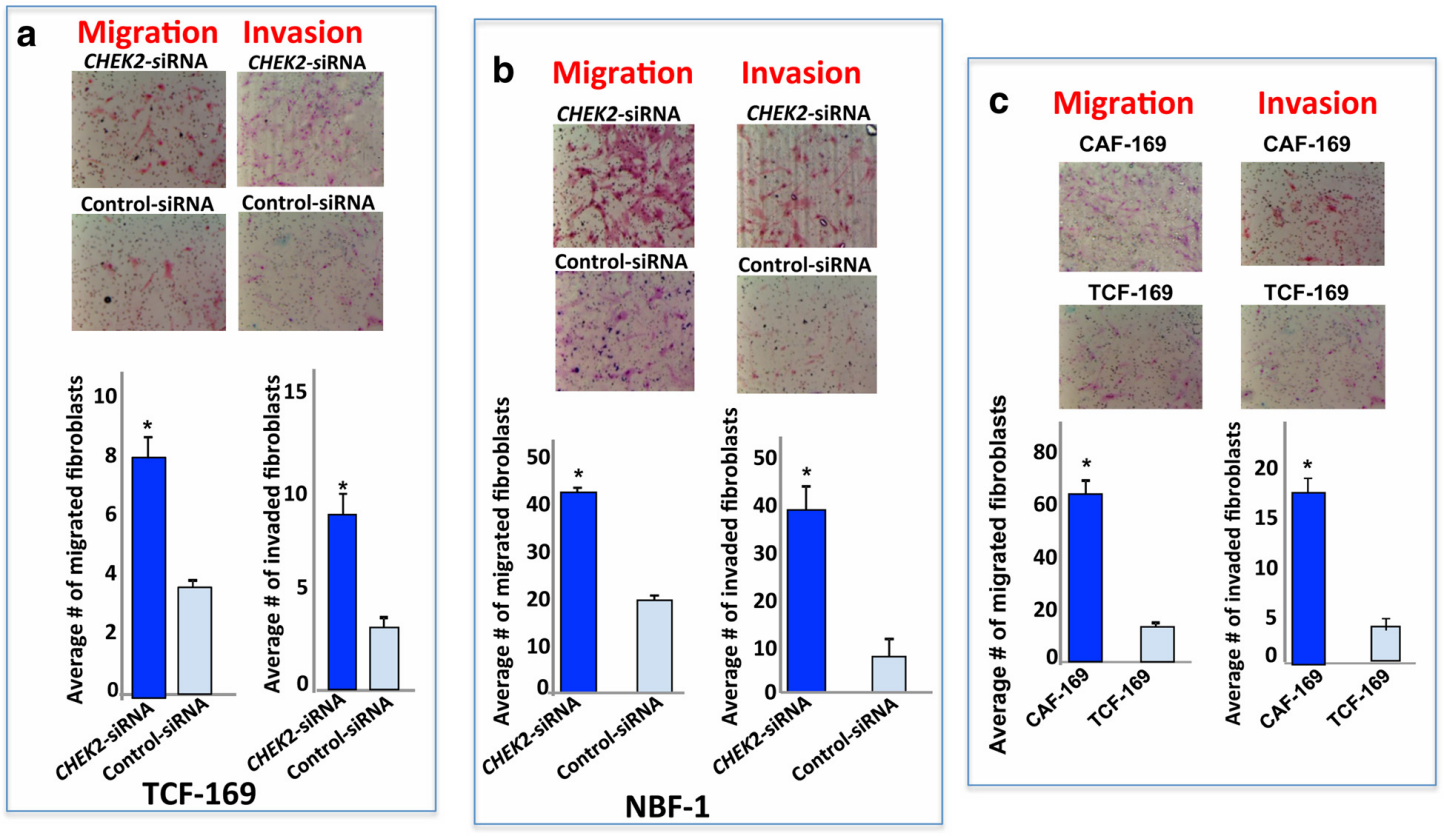
*CHEK2* represses the migration/invasion abilities of breast stromal fibroblasts. TCF-169-*CHEK2*-siRNA cells and their controls (**a**), NBF-1-*CHEK2*-siRNA cells and their controls (**b**) as well as CAF-169 and TCF-169 cells (**c**) were seeded with SFM in the inserts of BD bio-coat™ chambers (BD biosciences) (without matrigel, for migration) or coated with matrigel layer (for invasion), while complete media was added as a chemo-attractant to the lower wells of the chambers. After 24 h of incubation, cells were stained with Diff-Quick stain then counted. Upper panels: images of the invaded and migrated cells. Histograms: Average numbers of invaded and migrated cells. Error bars represent means ± S.D, *, *p* value < 0.0001

Next, we examined the effect of *CHEK2* down-regulation on the migratory and invasiveness abilities of stromal fibroblasts. To this end, TCF-169-*CHEK2*-siRNA cells as well as their respective controls were seeded with SFM in the upper inserts of BD bio-coat™ chambers (BD biosciences) (without matrigel, for migration) or coated with matrigel layer (for invasion), while complete media was added as a chemo-attractant to the lower wells of the chambers. Cells were then incubated overnight to allow migration and invasion. Figure 3a shows that TCF-169-*CHEK2*-siRNA cells were 2- and 3- fold more motile and invasive than their controls, respectively. Similar results were obtained when *CHEK2* was knocked-down using the same siRNA in the normal breast stromal fibroblasts NBF-1 (Fig. 3b). These results mirror the migration/invasion abilities of TCF-169 and their corresponding CAF-169 (Fig. 3c). This indicates that *CHEK2* represses the migration/invasion abilities of breast stromal fibroblasts.

### *CHEK2* down-regulation in breast stromal fibroblasts enhances the proliferation of epithelial cells in a paracrine manner

**Fig. 4:**
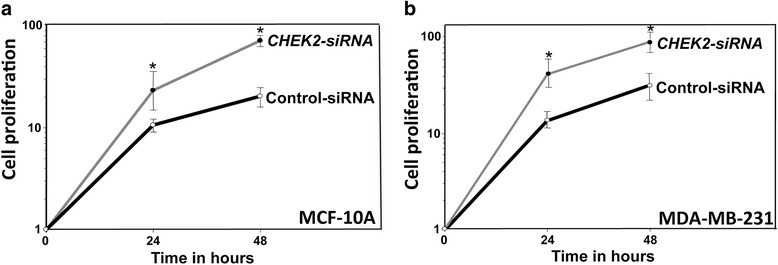
*CHEK2* deficient fibroblasts enhance the proliferation of epithelial cells in a paracrine manner. SFCM were collected from TCF-169-*CHEK2*-siRNA cells and their controls, and then were added to MCF10A (**a**) or MDA-MB-231 (**b**) cells that were seeded on 96-well plates. The proliferation was measured at the indicated times using the WTS-1 assay. Error bars represent ± SD, *p* value < 0.0001

To confirm the active status of *CHEK2*-defective fibroblasts, we tested the paracrine procarcinogenic effects of these fibroblasts. To this end, we first examined the effect of *CHEK2* down-regulation in stromal fibroblasts on the proliferation of epithelial cells utilizing indirect co-culturing. Therefore, SFCM was collected from TCF-169-*CHEK2*-siRNA cells and the corresponding control cells, and were used for culturing the non-cancerous MCF-10A and the cancer MDA-MB-231 epithelial cells seeded in 96 well plates. Cell proliferation was assessed using the WST-1 reagent. Figure 4 shows that after 24 h and 48 h of incubation, SFCM from TCF-169-*CHEK2*-siRNA enhanced cell proliferation as compared to SFCM from control cells, in both cell lines. This indicates that *CHEK2* down-regulation in breast stromal fibroblasts enhances their ability in increasing the proliferation of breast epithelial cells via paracrine signaling.

### *CHEK2* down-regulation in breast stromal fibroblasts promotes the migration/invasion abilities of breast cancer cells in an SDF-1/IL-6-dependent manner

**Fig. 5:**
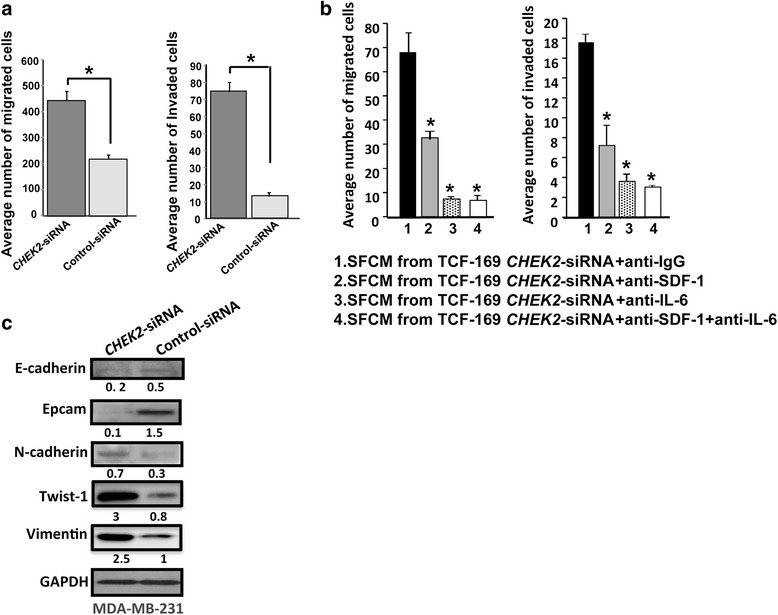
*CHEK2-*deficient fibroblast secretions stimulate invasion/migration in an SDF1- and IL-6-dependent manner and enhance mesenchymal features in breast cancer cells. **a** SFCM were collected after 24 h of incubation from TCF-169-*CHEK2-*siRNA and their control cells, and were added to MDA-MB-231 cells (10^5^) that were seeded onto the upper compartment of the plates and incubated for 24 h. The numbers of invaded and migrated cells were presented in histograms. Error bars represent means ± S.D. *: *p* value <0.001, as compared to the control lane 1. **b** MDA-MB-231 cells were treated as indicated, and then the migration and invasion abilities were assessed as described in A. **c** MDA-MB-231 cells were treated with SFCM from the indicated cells for 24 h, and then cell lysates were prepared and used for immunoblotting analysis using the indicated antibodies

We have next studied the effect of *CHEK2* down-regulation in stromal fibroblasts on the migration and invasion abilities of breast cancer MDA-MB-231 cells. Therefore, these cells were seeded in the upper inserts of the migration and invasion chambers in the presence of SFCM from TCF-169-*CHEK2*-siRNA and control cells, while the lower inserts of the chambers contained SFM. Cells were incubated overnight to allow migration and invasion under the attraction force of the chemo-attractants that are present in the SFCM. MDA-MB-231 cells that migrated/invaded were fixed, stained and counted in 5 random fields. Figure 5a shows that MDA-MB-231 cells that were exposed to SFCM from TCF-169-*CHEK2*-siRNA had higher migration/invasion activities as compared to cells exposed to SFCM from the corresponding control cells. Indeed, SFCM from *CHEK2*-deficient cells enhanced the migration and invasion abilities of MDA-MB-231 cells 2- and 7- fold as compared to control cells, respectively (Fig. 5a). These results provide evidence that *CHEK2* down-regulation in breast stromal fibroblasts increases the paracrine pro-migration and -invasion of cancer cells, which further confirms the active status of *CHEK2*-deficient breast stromal fibroblasts.

Next, we sought to investigate the possible role of SDF-1 and IL-6 in the paracrine effects of *CHEK2*-deficient cells. To this end, both cytokines were inhibited either separately or simultaneously using specific neutralizing antibodies in the SFCM from TCF-169-*CHEK2*-siRNA. Anti-IgG was also used as negative control. MDA-MB-231 cells were incubated with these media, and then the migration/invasion abilities of these cells were studied as described above. Figure 5b shows that the inhibition of SDF-1 reduced by 2 fold the migration potential of breast cancer cells as compared to cells exposed to anti-IgG. Interestingly, the inhibition of IL-6 was more potent, reducing the migration to a level similar to that obtained by the double inhibition (7 fold lower as compared to the control). In addition, the inhibition of SDF-1, IL-6 or both in SFCM collected from TCF-169-*CHEK2*-siRNA reduced the invasion potential of breast cancer cells 2.5, 5 and 6 fold, respectively (Fig. 5b). This shows that the paracrine IL-6 effect on the migration/invasion of breast cancer cells is stronger than that of SDF-1, and that the pro-migratory/-invasiveness effects of *CHEK2-*deficient stromal fibroblasts is mediated through increase in the secretion of SDF-1 and IL-6, which are in normal situations repressed by *CHEK2*.

### *CHEK2* down-regulation in breast stromal fibroblasts enhances the epithelial-to-mesenchymal transition of breast cancer cells in a paracrine manner

To further study the effect of *CHEK2* down-regulation on breast cancer cells, we investigated the paracrine effect of *CHEK2* deficient fibroblasts on the mesenchymal and epithelial markers of MDA-MB-231 cells. These cells were treated with SFM conditioned with TCF-169-*CHEK2*-siRNA or TCF-169-control-siRNA for 24 h. Subsequently, protein extracts were prepared and used for immunoblotting analysis using specific antibodies for EMT markers. Figure 5c shows that the levels of the epithelial markers E-cadherin and Epcam were reduced 2.3 and 12 fold in MDA-MB-231 cells treated with SFCM from TCF-169-*CHEK2*-siRNA as compared to SFCM from control cells. On the other hand, the levels of the mesenchymal markers N-cadherin, Twist1 and vimentin were 2.3, 12 and 3 fold higher in breast cancer cells treated with SFCM from TCF-169-*CHEK2*-siRNA as compared to SFCM from control cells (Fig. 5c). This indicates that SFCM from *CHEK2*-deficient fibroblast cells can enhance the EMT process in breast cancer cells.

### Ectopic expression of *CHEK2* suppresses the procarcinogenic features of cancer-associated fibroblasts

**Fig. 6:**
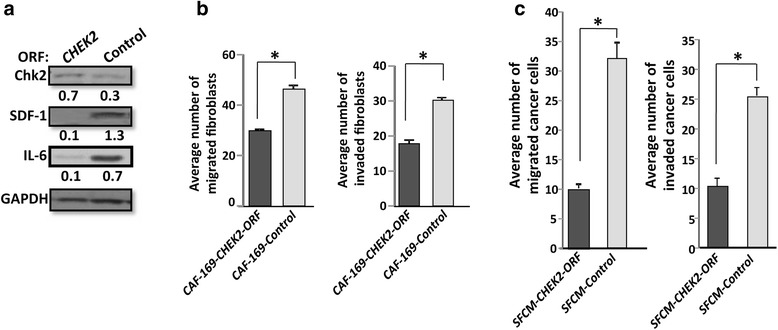
*CHEK2* upregulation represses breast stromal fibroblasts and their paracrine procarcinogenic effects*.* CAF-169 cells transfected with *CHEK2*-ORF (CAF-169-*CHEK2*-ORF) or control plasmid (CAF-169-control). **a** Whole cell lysates were prepared from the indicated cells and were used for immunoblotting analysis. The numbers below the bands indicate the corresponding expression levels after loading correction against GAPDH. **b** Assessment of the migration/invasion abilities of CAF-169 cells expressing either CHEK2 ORF or control plasmid as described in Fig. 3. **c** SFCM were collected after 24 h of incubation from CAF-169-*CHEK2*-ORF cells (SFCM-CHEK2-ORF) and control cells (SFCM-control), and then were added to MDA-MB-231 cells. The migration and invasion abilities were assessed as described in Fig. 5a. Error bars represent means ± S.D. *: *p* value <0.001

In an effort to further confirm the role of *CHEK2* in suppressing the procarcinogenic features of breast stromal fibroblasts, we next investigated the effects of *CHEK2* ectopic expression in CAF-169 cells, wherein *CHEK2* level was shown to be very low (Fig. 1a). Therefore, CAF-169 cells were transfected with a lenti-virus-based plasmid bearing *CHEK2*-ORF (CAF-169-*CHEK2*-ORF), and an empty vector was used as control. Whole cell lysates were prepared and used for immunoblotting analysis. Figure 6a shows that the Chk2 level was 2.3 fold higher in CAF-169-*CHEK2*-ORF cells as compared to the controls. Interestingly, ectopic expression of the *CHEK2* gene in CAF-169 cells strongly reduced the level of both SDF-1 and IL-6 (Fig. 6a). These levels mirror the SDF-1 and IL-6 levels observed in TCF-169 cells (Fig. 2b). This finding further supports the role of *CHEK2* in suppressing the expression of SDF-1 and IL-6. Next, we studied the migratory and the invasiveness abilities of CAF-169-*CHEK2*-ORF cells, using BD chambers. While complete medium was added as a chemo-attractant to the lower wells of the chambers, cells were added to the upper wells in the presence of SFM. Figure 6b shows that ectopic expression of *CHEK2* reduced the motility (40 %) and the invasiveness (34 %) of active CAF-169 cells as compared to control cells. This confirms that *CHEK2* represses the migration/invasion abilities in breast stromal fibroblasts.

To further show the role of *CHEK2* in suppressing the procarcinogenic effects of CAFs, we investigated the paracrine effect of *CHEK2* expression on the migration/invasion capacities of MDA-MB-231. Cells were seeded in the inserts of BD chambers, and SFM conditioned with CAF-169-*CHEK2*-ORF cells (SFCM-*CHEK2*-ORF) as well as control cells (SFCM-control) were placed in the lower wells of the chambers. Cells were incubated overnight to allow migration and invasion. Figure 6c shows that SFCM-*CHEK2*-ORF reduced the motility (3 fold) and the invasiveness (2.5 fold) of breast cancer MDA-MB-231 cells as compared to SFCM-control. This further confirms that *CHEK2* represses the paracrine pro-migration/invasion effects of breast stromal fibroblasts. Together, these results indicate that ectopic expression of the *CHEK2* gene in the active CAF-169 fibroblasts transforms these cells to a normal state.

## Discussion

Myofibroblasts actively contribute to the growth, expansion and dissemination of neoplastic epithelial cells. However, the genes and pathways controlling myofibroblast-related tumor-promoting effects are not fully delineated. Several lines of evidence indicate the implication of tumor suppressor genes in controlling the procarcinogenic effects of stromal fibroblasts in a paracrine manner [2, 3]. In the present study we have shown that the tumor suppressor Chk2 protein plays an important role in controlling mammary stromal fibroblast autocrine and paracrine signaling. Indeed, *CHEK2* is down-regulated in CAFs as compared to their corresponding TCFs, isolated from the same patients. In addition, we have seen inter-individual variation in the *CHEK2* expression among TCF cells. This could be due to different tumor features such as the stage or the grade. Similar differences were previously observed for p16 [3]. The *CHEK2* decrease suggests a role of cancer cells in suppressing *CHEK2* expression in fibroblast cells present in their vicinity, either directly or indirectly. In fact, several lines of evidence indicate that neoplastic cells have the ability to affect their microenvironment and modulate gene expression through secreted factors [17, 18]. This in principle leads to the activation of stromal cells, which in turn fuel the carcinogenic process and promote tumor growth and spread through functional cross-talk with tumor cells. To study the role of *CHEK2* down-regulation in these processes, we specifically down-regulated this gene in breast stromal fibroblasts using specific siRNA, and we studied the consequent autocrine and paracrine effects. We have shown that *CHEK2* down-regulation activates breast stromal fibroblasts and enhances their paracrine procarcinogenic effects. Indeed, *CHEK2*-defective cells exhibited higher migratory and invasive capacities, expressed lower levels of p53 and higher levels of the myofibroblast markers α-SMA and SDF-1, and enhanced the migration/invasion abilities of breast cancer cells and their mesenchymal features in a paracrine manner. Indeed, *CHEK2* down-regulation in fibroblasts paracrinaly induced the three major mesenchymal markers N-cadherin, Twist-1 and vimentin, and decreased two major epithelial markers E-cadherin and Epcam in breast cancer cells. This indicates that *CHEK2*-defective breast stromal fibroblasts enhance the pro-metastatic EMT process in epithelial cells in a paracrine manner. On the other hand, ectopic expression of *CHEK2* repressed the migratory/invasiveness abilities of active breast stromal fibroblasts as well as their paracrine effects on breast cancer cells. This confirmed the suppressive role of *CHEK2* in the procarcinogenic effects of breast stromal fibroblasts.

The present data showed also that in addition to its autonomous tumor suppressor function, *CHEK2* has also a non-cell-autonomous tumor suppressor activity. This paracrine effect is mediated through controlling the expression/secretion of the pro-carcinogenic cytokines SDF-1 and IL-6, may be through p53 down-regulation. Indeed, p53 represses the expression of both SDF-1 and IL-6 [19, 20], which are important promoters of tumor growth and progression [11, 12]. Furthermore, specific inhibition of secreted SDF-1 or IL-6 suppressed the pro-migratory/invasiveness effects of *CHEK2*-deficient stromal fibroblasts. Interestingly, the double blockage had an inhibitory effect similar to that obtained by the inhibition of IL-6 alone, which was stronger than SDF-1 inhibition (Fig. 5). This indicates that the effects of IL-6 and SDF-1 inhibition were synergistic despite the fact that the 2 molecules act through different receptors and pathways, however, a potential functional interaction between the activated pathways is possible.

In addition to Chk2, other important tumor suppressor proteins such as p16, p21, p53, PTEN and CAV-1 are also implicated in repressing the procarcinogenic effects of breast stromal fibroblasts both in vitro and in vivo [2, 3, 19, 21–24]. This clearly shows that these tumor suppressor proteins represses breast carcinogenesis in both epithelial as well as stromal cells.

## Conclusions

The present findings have shown that in addition to its well-known cell-autonomous tumor suppressor function within incipient pro-carcinogenic epithelial cells, stromal fibroblast *CHEH2*, like other tumor suppressor genes, exerts also non-cell-autonomous effects in breast tumorigenesis. Therefore, stromal fibroblast *CHEK2* might constitute a valid therapeutic target to stop tumor progression and/or recurrence.

## Abbreviations

ATCC, American type culture collection; DMSO, dimethyl sulfoxide; EMT, epithelial-to-mesenchymal transition; GAPDH, glyceraldehyde-3-phosphate dehydrogenase; ORF, open reading frame; PBS, phosphate buffered saline; RT-PCR, reverse transcriptase-polymerase chain reaction; siRNA, small interfering RNA
